# Adiposopathy and bariatric surgery: is ‘sick fat’ a surgical disease?

**DOI:** 10.1111/j.1742-1241.2009.02151.x

**Published:** 2009-09

**Authors:** H E Bays, B Laferrère, J Dixon, L Aronne, J M González-Campoy, C Apovian, B M Wolfe, The adiposopathy and bariatric surgery working group

**Affiliations:** 1 Louisville Metabolic and Atherosclerosis Research CenterLouisville, KY, USA; 2 Department of Medicine, Endocrine, Diabetes and Nutrition Division, New York Obesity Research Center, Columbia University College of Physicians and Surgeons, St Luke’s Roosevelt Hospital CenterNew York, NY, USA; 3 Department of General Practice, Obesity Research Unit, Monash UniversityMelbourne, Vic., Australia; 4 Neurotransmitter Laboratory, Baker IDI Heart and Diabetes InstituteMelbourne, Vic., Australia; 5 Clinical Professor of Medicine, Weill-Cornell Medical College, Director of the Comprehensive Weight Control Program, Cornell UniversityNew York, NY, USA; 6 Minnesota Center for Obesity, Metabolism & Endocrinology (MNCOME)Eagan, MN, USA; 7 88 East Newton Street, Building D Suite 614, Boston, MA, USA; 8 General Surgery, Oregon Health & Science UniversityPortland, OR, USA

## Abstract

**Objective:**

To review how bariatric surgery in obese patients may effectively treat adiposopathy (pathogenic adipose tissue or ‘sick fat’), and to provide clinicians a rationale as to why bariatric surgery is a potential treatment option for overweight patients with type 2 diabetes, hypertension, and dyslipidaemia.

**Methods:**

A group of clinicians, researchers, and surgeons, all with a background in treating obesity and the adverse metabolic consequences of excessive body fat, reviewed the medical literature regarding the improvement in metabolic disease with bariatric surgery.

**Results:**

Bariatric surgery improves metabolic disease through multiple, likely interrelated mechanisms including: (i) initial acute fasting and diminished caloric intake inherent with many gastrointestinal surgical procedures; (ii) favourable alterations in gastrointestinal endocrine and immune responses, especially with bariatric surgeries that reroute nutrient gastrointestinal delivery such as gastric bypass procedures; and (iii) a decrease in adipose tissue mass. Regarding adipose tissue mass, during positive caloric balance, impaired adipogenesis (resulting in limitations in adipocyte number or size) and visceral adiposity are anatomic manifestations of pathogenic adipose tissue (adiposopathy). This may cause adverse adipose tissue endocrine and immune responses that lead to metabolic disease. A decrease in adipocyte size and decrease in visceral adiposity, as often occurs with bariatric surgery, may effectively improve adiposopathy, and thus effectively treat metabolic disease. It is the relationship between bariatric surgery and its effects upon pathogenic adipose tissue that is the focus of this discussion.

**Conclusions:**

In selective obese patients with metabolic disease who are refractory to medical management, adiposopathy is a surgical disease.

**What’s known**

Bariatric surgery is indicated to treat obese patients, especially those with comorbidities.

**What’s new**

Bariatric surgery is not only effective in reducing fat mass, but also effective in improving many metabolic diseases associated with obesity through correction of adiposopathy (“sick fat”) as well as possibly through mechanisms independent of its effects upon adipose tissue.

## Introduction

Adiposopathy (“sick fat”) is defined as pathogenic adipose tissue that is promoted by positive caloric balance, increased energy storage and sedentary lifestyle in genetically and environmentally susceptible patients (1). Impaired adipocyte proliferation or differentiation (adipogenesis), visceral adiposity, growth of adipose tissue beyond adequate vascular supply and ectopic fat deposition are anatomical manifestations of adiposopathy that are associated with adverse endocrine and immune responses leading to metabolic disease (2,3).

Adipose tissue is an active endocrine organ (4–6) and an active immune organ (7,8). Its functionality is important for metabolic health. During positive caloric balance, the manner in which energy is stored correlates to the risk of developing metabolic disease. For example, adipogenesis is a biological process involving proliferation of preadipocytes from precursor cells, and then their differentiation into mature adipocytes. Adipocytes and adipose tissue express a number of factors that *directly* influence adipogenesis (such as modulating and transcription factors) as well as factors that *indirectly* influence adipogenesis (such as those that affect angiogenesis, as well as the dissolution and reformation of adipose tissue extracellular matrix) (9). It is the unencumbered ability of adipocytes to proliferate and differentiate during positive caloric balance that prevents adipocyte dysfunction. This is clinically important because impaired adipocyte proliferation (resulting in excessive fat cell enlargement) and impaired adipocyte differentiation may both cause adipocyte dysfunction, which increases the risk of metabolic disease (3,9). For example, if storage of fats in adipose tissue is impaired during positive caloric balance due to inadequate adipogenesis, then this may result in an ‘overflow’ of lipid and free fatty acids (10). Increases in circulating free fatty acids may be ‘lipotoxic’ to various organs, which contribute to metabolic disease (10). Thus, adipogenic factors produced by adipocytes/adipose tissue (as well as other body organs) are an important determinant of whether an increase in adipose tissue simply increases the risk of adipose tissue mass pathology, or increases the risk of adipose tissue metabolic pathology as well.

Previously, a group of clinicians and researchers, each with a background in endocrinology, assembled together and reached a consensus that: *Adiposopathy (“sick fat”) is an endocrine disease* (11). This reflected the recognition that pathogenic adipose tissue can ‘cause’ and/or worsen metabolic disease. From a clinical standpoint, it is equally relevant that therapeutic interventions that reduce adipose tissue’s adverse endocrine and immune responses may ‘cure’ or improve metabolic disease (12,13).

Examples of interventions that may improve the adverse metabolic consequences of adiposopathy include appropriate nutrition, increased physical activity and pharmaceuticals (12). Bariatric surgery in obese patients is also an intervention that reduces body fat and effectively treats type 2 diabetes mellitus (T2DM) (14–16), hypertension (14,16) and dyslipidaemia (16). Current evidence supports that the improvement in metabolic diseases with bariatric surgery is largely through reversing the otherwise pathogenic adipose tissue endocrine and immune responses associated with pathogenic adipose tissue. The efficacy of bariatric surgery on metabolic disease can be dramatic with 30–60%‘resolution’ of high blood pressure (17,18) and 70% improvement in dyslipidaemia (18). Most notable is the remission of T2DM with bariatric surgery, which is reported to occur as high as ∼80% with gastric bypass (17,18).

This follow-up adiposopathy consensus paper reviews the effects of bariatric surgery on adipose tissue pathophysiology, as well as its therapeutic effects upon metabolic diseases often associated with ‘sick fat’.

## Surgical treatments for adiposopathy

Historically, surgical obesity treatments have utilised a variety of techniques (19). Currently, the most common bariatric surgeries include gastric bypass and laparoscopic adjustable gastric banding (LAGB). These different surgical procedures may produce different metabolic results.

### Gastric bypass

Gastric bypass, through the creation of a Roux-en-Y anastomosis, was named after Cesar Roux, a Swiss surgeon. The Roux-en-Y surgery was originally performed in 1892 for the intent of ‘bypassing’ the stomach to treat antral or pyloric obstruction (20). Since the 1950s and 1960s, the gastric bypass procedure has evolved as a treatment for obesity (21,22). It commonly involves creation of a small (20–30 cc) proximal stomach pouch, attached to the distal end of the oesophagus. This is achieved by stapling the proximal stomach, and then surgically dividing the partitioned gastric pouch from the rest of the stomach. The next step is the division of the jejunal portion of the small intestine into a jejunal proximal limb (which maintains its attachment to the duodenum), and a jejunal distal ‘Roux limb,’ which is then connected (anastomosis) to the newly created stomach pouch (gastrojejunostomy) (19). Finally, the residual proximal jejunal limb (attached to the duodenum and stomach) undergoes anastomosis at a variable distance distal from the jejunal division. This jejunojejunostomy completes the ‘Y’ configuration. With this procedure, ingested nutrients pass directly from the gastric pouch into the jejunal Roux limb of the jejunum. Gastric, pancreatic and biliary fluids continue to drain through the residual jejunal proximal limb that remains attached to the duodenum/stomach, which then empties into a more distal jejunal location. Thus, ingested nutrients ‘bypass’ most of the stomach, and ‘bypass’ all of the duodenum and proximal jejunum, resulting in more distal digestion.

The length of each limb may vary according to surgical technique and preference. With a standard gastric bypass procedure (sometimes termed proximal gastric bypass), the risk of malabsorption of macronutrients is minimal. However, with a distal gastric bypass procedure, the jejunal severing is more distal from the stomach. Less small intestine then remains in the roux limb and more of the jejunum is bypassed. While some reports suggest that proximal vs. distal gastric bypass do not differ with regard to efficacy and complications (23), it is the experience of others that distal gastric bypass may result in greater weight loss. However, it may also increase the risk of macronutrient malabsorption. Thus, it is unclear if the potential, yet un-established benefits of additional weight loss from distal gastric bypass procedures warrant the increased potential metabolic risks of malabsorption. As such, some surgeons are very selective about choosing to perform the distal gastric bypass procedure, with others preferring not to perform this type of procedure at all (24).

Gastric bypass has several potential mechanisms accounting for its efficacy. The small size of the newly created gastric pouch may restrict the volume of food intake, and alters endocrine and neural signalling of appetite and satiety. As noted above, depending upon the surgical technique, malabsorption of macronutrients may sometimes contribute to weight loss efficacy, particularly with the less commonly used distal gastric bypass procedure. Regarding micronutrients, malabsorption can occur with any gastric bypass procedure, and may result in nutritional deficiencies (Table 1). Finally a ‘dumping syndrome’ (25) may occur, which is caused by rapid expansion of the jejunum with hyperosmolar food. Dumping syndrome is clinically manifest by nausea, vomiting, bloating, cramping, diarrhoea and fatigue immediately after a meal or as long as 3 h after a meal.

**Table 1:** Laparoscopic adjustable gastric banding (LAGB) vs. gastric bypass (16,108,130)

	**LAGB**	**Gastric bypass**
Common characterisation of procedure	Restrictive*	Multimechanistic†
Weight loss (mean)	Gastric bypass > LAGB (∼20–30 kg)	Gastric bypass > LAGB (∼30–40 kg)
Improvement of obesity-related comorbidities	Gastric bypass > LAGB	Gastric bypass > LAGB
Short-term morbidity	Gastric bypass > LAGB	Gastric bypass > LAGB
Perioperative mortality (131)	Gastric bypass > LAGB	Gastric bypass > LAGB
Reoperation and readmission	Variable (∼5–10%)	Variable (∼5–10%)
Days of hospitalisation for procedure	1 Day or less for procedure, if no complications	2–4 Days for open procedure
Able to be performed laparoscopically	Yes	Yes
Permanent alterations in gastrointestinal tract	No, band may be adjusted or removed	Yes, although ‘reversal’ reoperation can be performed
Loss of fat-free mass (132)	Gastric bypass > LAGB	Gastric bypass > LAGB
2008 Initial cost estimate	$17,000	$26,000
Acute complications	Band too tight with gastrointestinal obstructive symptoms Haemorrhage Gastrointestinal bleeding Infection Cardiac dysrhythmias Atelectasis and pneumonia Deep vein thrombosis	Gastrointestinal obstruction Haemorrhage Gastrointestinal bleeding Anastomotic leaks Infection Cardiac dysrhythmias Atelectasis and pneumonia Deep vein thrombosis Pulmonary emboli Rhabdomyolysis
Chronic complications (22)	Band slippage, erosion, port infection, disconnection and displacement Oesophageal dilation Rare nutrient deficiencies if persistent vomiting or marked and sustained decrease in nutritional intake Unclear effects on depression (133,134)	Marginal ulcers Oesophageal dilation Dumping syndrome with reactive hypoglycaemia Small bowel obstruction caused by internal hernias or adhesions Anastomotic stenoses (stomal narrowing) Gallstones Calcium deficiency Secondary hyperparathyroidism Iron deficiency Protein malnutrition Other nutritional and mineral deficiencies (e.g. deficiencies of vitamins A, C, D, E, B, and K, folate, zinc, magnesium, thiamine, etc.) (135) Anaemia (often related to mineral and nutrition deficiencies) Metabolic acidosis Bacterial overgrowth Kidney stones (oxalosis) Neuropathies (resulting from nutritional deficiencies) Osteoporosis (often caused by calcium deficiency and chronically elevated parathyroid hormone levels) Improvement or worsening of depression (133,136)
Need for long-term follow-up	LAGB = Gastric bypass	LAGB = Gastric bypass

* While often characterised as a ‘restrictive’ procedure, macronutrient transit may not be delayed with LAGB, and the weight loss effects may be the result of increased satiety and decreased hunger (see text).

† The Roux-en-Y gastric procedure has a combination of both restrictive and malabsorptive elements (23,27), and thus its mechanisms of weight loss ad metabolic efficacy are complex.

### Laparoscopic adjustable gastric banding (LAGB)

In the 1970s, non-adjustable banding around the upper part of the stomach emerged as a bariatric surgical procedure (26). In the early 1980s, adjustable gastric bands were developed, and in the 1990's, underwent more widespread clinical use. With this procedure, a silicone band is laparoscopically placed around a proximal portion of the stomach. The band diameter can be adjusted. No division or anastomosis of the intestine is involved. If the band is too tight, the transit of food from the oesophagus and proximal stomach into the rest of the stomach may be impaired, potentially resulting in the unintended complication of postprandial vomiting and need for gastric band adjustment. However, while LAGB is often described as a ‘restrictive’ procedure (with gastric bypass also having a ‘restrictive’ component) (27) (Table 1), the weight loss efficacy of this ‘restrictive’ procedure, in most cases, may not be due to delayed nutrient flow. Gastric emptying does not appear to be altered by LAGB (28). Some suggest that the predominant mechanisms accounting for LAGB weight loss efficacy are increased satiety (29) and decreased hunger (30).

## Effects of bariatric surgery upon adiposopathy

### Adipose tissue anatomical changes with bariatric surgery

It may be intuitive to expect that the greatest long-term effects of bariatric surgery upon adiposopathy would be related to effects upon adipose tissue anatomy and physiology. When bariatric surgery results in weight loss in patients with excessive fat-related metabolic disease, a reduction in adipocyte size (31) and a reduction in visceral adiposity (32) would both be expected to result in improvements in adipose tissue endocrine and immune responses. Thus, by favourably altering adipose tissue anatomy from both a cellular and organ standpoint, bariatric surgery improves metabolic disease (9,13,33).

On a cellular level, excessive adipocyte hypertrophy leads to fat cell dysfunction that might be characterised as representing ‘sick fat’ (1). The ‘sick fat’ associated with excessive fat cell enlargement represents a ‘stress condition’ for intracellular organelles such as the endoplasmic reticulum (34), which can lead to inflammation, insulin resistance, limitations on energy storage, net increase in circulating free fatty acids (35), lipotoxicity (10), and adverse endocrine and immune responses. All of these pathogenic adipose tissue effects contribute to metabolic diseases such as T2DM (2,9). On an organ level, adipose tissue growth beyond its vascular supply is an anatomical manifestation of adiposopathy that may result in hypoxia (9). Hypoxia promotes inflammatory responses that are increasingly being recognised as a potential contributor to metabolic disease (3,36,37). Some authors have suggested that *hypoxia may underlie the inflammatory response in adipose tissue* (38). Thus, a reduction in fat cell size and reduction in adipose tissue growth beyond its vascular supply are favourable effects that at least partially explain the observed reduction in inflammatory markers with a reduction in adiposity, as occurs with bariatric surgery.

Another anatomical finding with bariatric surgery is a reduction in hepatic fat (steatosis) (39,40), with the possible exception of those who lose weight very rapidly (41). Reduced hepatic steatosis with bariatric surgery is an anatomic finding that may reflect a decrease in free fatty acid delivery to the liver (as a result of improved ability of adipose tissue to store circulating free fatty acids) and a diminished potential for lipotoxicity (10). As with other anatomic findings with fat weight loss, a reduction in hepatosteatosis may be associated with an improvement in metabolic disease (9,13).

### Bariatric surgery and the six faces of adiposopathy

Pathophysiologically, adiposopathy has at least six ‘faces’ that contribute to metabolic disease (13). Briefly, bariatric surgery may affect these biological processes in the following ways:

(1) *Impaired adipogenesis*: Adipogenesis is a biological process necessary for optimal storage of excess energy during positive caloric balance. Energy storage (a critical function of adipose tissue) is dependent upon adipocyte number and size (3), and is determined by the proliferation of precursor cells in to preadipocytes, which then undergo differentiation into mature adipocytes (42). During positive caloric balance, if calories are stored through adequate adipogenesis, then the patient may not manifest metabolic disease, even with fat weight gain. Conversely, if fat cell proliferation is inadequate during positive caloric balance, then excessive adipocyte hypertrophy may ensue, which is associated with pathogenic endocrine and immune responses that contribute to metabolic diseases (9). If fat cell differentiation is impaired, then adipocyte storage of energy may also be impaired, resulting in pathogenic adipose tissue responses as well (43).

Data are relatively scarce regarding the effect of bariatric surgery upon the numerous adipose tissue and non-adipose tissue adipogenic factors (e.g. those involved with adipocyte modulation and transcription, extracellular matrix formation, angiogenesis, etc.) (9). Ghrelin (derived from ‘gh’ for growth hormone and ‘relin’ for release) is an orexigenic peptide hormone whose secretion increases before meals, and decreases after meals. In addition to stimulating the central growth-hormone secretagogue receptor 1a (44), ghrelin may also stimulate cell proliferation and inhibit preadipocyte differentiation (45). Because it originates from the stomach, ghrelin may be an important hormone influenced by bariatric surgery. However, the reported effects of bariatric surgery upon ghrelin levels are inconsistent, with at least one study suggesting that fasting ghrelin is inversely related to body mass index (BMI), with no apparent independent effect of gastric bypass on ghrelin levels beyond weight loss alone (46). Therefore, it is as yet unclear that ghrelin levels alone provide clinically meaningful information, such as predicting weight loss in patients undergoing bariatric surgery (47).

Adiponectin is an anti-inflammatory protein secreted from adipocytes whose secretion is often decreased with obesity, insulin resistance and T2DM (2). Adiponectin may promote cell proliferation and differentiation of preadipocytes into adipocytes (48). Bariatric surgery resulting in weight loss is associated with increased adiponectin levels (49).

Another example of an adipogenic marker that may be altered with bariatric surgery is acylation-stimulating protein, which enhances short-term energy clearance and fat storage through increasing triglyceride (TG) synthesis in fat cells (2). This lipogenic adipocyte protein increases with adipocyte differentiation and decreases after bariatric surgery (50).

Although data such as these are limited, some preliminary hypotheses might reasonably be made based upon the above. During weight loss, the ability or capacity for adipogenesis may or may not be increased with weight loss (51), and some pro-adipogenic adipoctye factors may actually increase. However, as long as weight loss is ongoing, the net fat cell proliferation signalling may be decreased; lipogenic factors may be decreased as well. A decrease in adipogenic markers with bariatric surgery may be a normal physiological response to the reduced caloric balance after bariatric surgery; because with less caloric intake, less adipogenic signalling is required for energy storage. This would help explain why fat cell number is decreased in patients losing weight after bariatric surgery (52). Other data also support that a decrease in adipogenesis with weight loss may be physiological, and not pathophysiological. For example, bariatric surgery may improve metabolic markers such as insulin and adiponectin, while simultaneously decreasing adipogenic markers such as vascular endothelial growth factor-A (53).

(2) *Increased pathogenic responses of adipose tissue storage depots most described to promote metabolic disease*: While hypertrophy of multiple adipocyte depots may contribute to metabolic disease, visceral fat is among the most metabolically active adipose tissue depots (9). Visceral fat is the best described depot wherein an increase in its mass results in pathogenic adipose tissue endocrine and immune responses. Visceral adiposity is associated with an increased release of pathogenic factors into the portal circulation leading directly to the liver (13). Thus, visceral adiposity contributes to hepatic-mediated metabolic diseases, such as T2DM and dyslipidaemia (9). Through a global reduction in adiposity, bariatric surgery reduces visceral adipose tissue (32), which may help to account for its efficacy in reducing the metabolic disease.

(3) *Increased circulating free fatty acids*: A net increase in circulating free fatty acids occurs when adipocyte lipolysis exceeds adipocyte lipogenesis, as occurs with adiposopathy (9). Increased circulating free fatty acids may contribute to lipotoxicity (10) as well as fatty liver (hepatosteatosis). However, not all free fatty acids contribute to lipotoxicity (54) (e.g. polyunsaturated fatty acids such as omega-3 fatty acids) (55). It is also unclear if increased hepatic TG content, so often found in overweight patients with metabolic disease, is simply a marker for increased free fatty acid delivery to the liver, or if hepatic TGs themselves actively participate in creating derangements leading to metabolic disease. What does seem to be clear is that an increase in circulating saturated free fatty acids increases the flux of free fatty acid to body organs. This may result in lipotoxicity to the liver (causing insulin resistance and increased hepatic glucose output), pancreas (causing impaired beta cell function with relative insulinopenia) and muscle (with the accumulation of intramyocellular lipids, such as diacylglycerol, fatty acyl CoA and ceramides also contributing to insulin resistance) (3,9,10).

Bariatric surgery can sometimes result in rapid weight loss, as might occur with bariatric procedures associated with significant malabsorption. If so, then similar to periods of starvation or during administration of very low calorie diets (56), free fatty acids may initially be increased for the first month or so, as result of rapid lipid mobilisation from fat depots (57). However, presumably after stabilisation of fat weight loss, circulating free fatty acids decrease (58,59). A reduction in circulating free fatty acids may contribute to the reduction in metabolic disease after bariatric surgery.

(4) *Pathogenic adipose tissue endocrine responses*: An increase in body fat, particularly when associated with adipocyte hypertrophy and visceral adiposity, causes pathogenic adipose tissue endocrine responses that contribute to metabolic diseases (3). The mechanisms by which these endocrine abnormalities contribute to metabolic disease are beyond the scope of this discussion (2,9). However, Table 2 shows the reported effects of pharmaceutical and surgical interventions upon adipose tissue endocrine factors that are among the most commonly described to contribute to T2DM, hypertension and dyslipidaemia.

**Table 2:** Examples of treatments for adiposopathy and their effects upon illustrative and selected adipose tissue factors that may contribute to metabolic disease (11–13). Medical and surgical therapies that treat adiposopathy result in improvement in multiple adipose tissues metabolic parameters, which helps explain why these same treatments improve T2DM, hypertension and dyslipidaemia

	May affect glucose metabolism, blood pressure and lipid metabolism				May affect glucose metabolism	May affect blood pressure	May affect lipid metabolism	
Intervention	Visceral adipose tissue	Free fatty acids	Leptin	Adiponectin	Tumour necrosis factor-α	Renin– angiotensin– aldosterone enzymes	Androgens	Oestrogens
Nutrition and physical activity	↓	↓	↓	↑	↓	↓	↓ (women) ↑ (men)	↓/– (men)
PPAR-γ*agonists (pioglitazone, rosiglitazone)	↓/–	↓	↓/–	↑	↓	–	↓	↓/– (men)
Orlistat	↓	↓	↓	↑	↓	?	↓ (women)	?
Sibutramine	↓	↓	↓	↑/–	?	?	↓ (women)	?
Cannabinoid receptor antagonists†	↓	↓	↓	↑	↓	?	?	?
LAGB§	↓ (137)	?	↓(29)	↑(138)	↓ (60) (130)	?	?	?
Gastric bypass§	↓ (139)	↓ (59)¶	↓(33)	↑(140)	↓ (140) (130)	↓ (141)	↑ (men) (142)	↓ (men) (142)

↑, increased; ↓, decreased; ?, unreported; –, neutral effect. LAGB, laparoscopic adjustable gastric banding; PPAR-γ, peroxisome proliferator–activated receptor-γ; T2DM, type 2 diabetes mellitus. *PPAR gamma agents may: (i) increase adipose tissue proliferation and differentiation, (ii) favourably alter the visceral to subcutaneous adipose tissue deposition ratio, (iii) reduce hepatic fat deposition, and (iv) have improve other aspects of adipose tissue function (10,12,13). While some of the weight gain associated with PPAR gamma agents results from fluid retention, much of the weight gain observed with these agents used to treat metabolic diseases traditionally associated with fat weight gain, paradoxically results from promoting increased amounts of functional adipose tissue. †Not available in US. §Less weight loss with LABG, compared with gastric bypass, may be associated with less pronounced improvements in inflammatory markers (130).¶Acutely, (e.g. 1 month) free fatty acids may be increased (see text).

(5) *Pathogenic adipose tissue immune responses*: An increase in adipocyte hypertrophy and visceral adiposity causes pathogenic adipose tissue immune responses that contribute to metabolic diseases (3). The mechanisms by which adiposopathic immune abnormalities contribute to metabolic disease are beyond the scope of this discussion (2,9). However, Table 2 shows the reported effects of pharmaceutical and surgical interventions upon immune factors that are among the most commonly described to contribute to T2DM, hypertension and dyslipidaemia.

Bariatric surgery may also have unique immunological considerations, compared with other therapeutic interventions. The more invasive the surgical procedure, and the more need for subsequent healing, then the greater the expected degree of pro-inflammatory markers following bariatric surgery. A subsequent reduction of inflammatory markers, many of which also originate from pathogenic adipose tissue, may take as long as 3–6 months after bariatric surgery (33,60). Even with consideration of gastric bypass alone, the systemic ‘stress’ and immune response may depend on how the procedure is performed. For example, the pro-inflammatory response from an open gastric bypass may be greater than with a laparoscopic gastric bypass, suggesting that the open procedure has a greater degree of operative injury (61). Nonetheless, after the transient increase in inflammatory markers have abated over time, significant and sustained weight reduction may be required to reduce long-term pathogenic adipose tissue inflammatory responses, which may otherwise contribute to metabolic disease (2).

(6) *Pathogenic interactions or ‘cross-talk’ with other body organs*: The promotion of metabolic disease associated with adiposopathy is substantially dependent upon the interaction or ‘cross-talk’ with multiple other body organs, such as the nervous system, immune system, skeletal muscle, cardiovascular system, liver, gastrointestinal system, adrenal cortex and thyroid (9). In patients with metabolic impairments of other body organs, even mild to moderate pathogenic adipose tissue endocrine and immune responses may overwhelm the ability of these other organs to compensate, which then contributes to metabolic disease (9). Conversely, in patients without impairment in other body organs, even if excessive body weight persists after bariatric surgery, the pathogenic responses associated with adiposopathy may become reduced to levels that are ‘manageable’ by other body organs (62).

## Mechanisms of improvement in metabolic disease with bariatric surgery beyond weight loss

Bariatric surgery has potential mechanisms independent of fat weight loss that may improve metabolic disease. Gastrointestinal surgery, such as bariatric surgery, requires acute fasting, which results in metabolic effects that may improve metabolic disease. Acute withdrawal of caloric intake (such as through fasting/starvation) may actually worsen insulin sensitivity and glucose tolerance in lean and obese individuals with and without T2DM, for at least as long as 60 h. However, as with any dramatic reduction in caloric intake in patients with diabetes mellitus, hypoglycaemic medications must be withheld or reduced to avoid the risk of hypoglycaemia before and immediately after bariatric surgery. Thus, if the procedure of fasting in the short, peri-operative phase results in less need for anti-diabetes mellitus drug treatment and if a reduction in the need of anti-diabetes mellitus drug treatment is considered a marker for improved glucose metabolism, then acute, short-term pre, peri and postoperative fasting might be argued as an illustrative example by which bariatric surgery might acutely improve metabolic disease. Similarly, acute fasting may also result in short-term (< 1 week) increased circulating lipids such as cholesterol and TG levels (63). Conversely, because of the lack of caloric intake, postprandial gastrointestinal lipid absorption does not occur, and thus postprandial lipaemia is reduced. Regarding blood pressure, water-only fasting may reduce blood pressure in hypertensive patients (64). Given all the above, the effects of peri-operative fasting on metabolic disease parameters might best be considered to be mixed. However, it is unlikely that acute peri-operative fasting significantly contributes to the longer-term effects of bariatric surgery upon metabolic disease.

Another manner in which bariatric surgery might favourably affect metabolic disease (and obesity itself) (65) is through altering an array of endocrine and immune responses from the gastrointestinal tract (66,67). As with adipose tissue, the gastrointestinal system is an active endocrine and immune organ. It is through the alteration in the secretion of gastrointestinal hormones that has led some to postulate that T2DM may be a surgical disease (68). Examples of proposed mechanisms as to how bariatric surgery may contribute to weight loss and improve metabolic disease beyond its favourable effects on adipose tissue include: (i) the ‘lower intestinal hypothesis,’ wherein rapid delivery of nutrients to the distal bowel enhances the secretion of incretins such as glucagon-like peptide and glucose-dependent insulinotropic peptide (i.e. gastric inhibitory peptide) (69–72) as well as non-incretin peptides such as Peptide YY (27) and (ii) the ‘foregut exclusion theory’ wherein bypassing the duodenum and proximal jejunum results in putative signalling favourable to glucose metabolism (27,73). However, not all data support that improvements in metabolic disease (or even weight loss) are caused by the alterations in gastrointestinal tract hormone and immune alterations found with bariatric surgery (74,75). Furthermore, while it is unclear the extent to which these gastrointestinal endocrine and immune responses contribute to improvement in glucose sensitivity and pancreatic beta cell function, it does appear that caloric restriction and weight loss are the dominant mechanisms by which bariatric surgery improves overall glucose metabolism (76).

## Responses to challenges and claims regarding bariatric surgery as a treatment for metabolic diseases

Based upon the authors’ experiences in prior publication efforts, review of the medical literature, as well as discussions at scientific meetings and expert conferences, the following claims listed below are among the more common challenges to bariatric surgery as a treatment for metabolic disease.

### Challenge #1: Bariatric surgery has insufficient published scientific information warranting its use

*Claim*: Not enough is known about the metabolic effects of current bariatric procedures, and thus not enough is known about potential mechanisms of action as to why bariatric surgery may improve metabolic disease.

*Response*: It is true that some of the more common bariatric surgery procedures, such as LAGB, have limited published data on some of the more commonly measured adipose tissue endocrine and immune markers thought to contribute to metabolic disease (Table 2). But more important than the need to better understand the intricate mechanisms as to *why* bariatric surgery has potential health benefits is the need to know that it *does* have potential health benefits for obese patients with metabolic disease. The facts are that weight gain is clearly associated with an increased risk of metabolic disease (2,62,77), and that the clinical trials data strongly support bariatric surgery as among the most effective therapeutic interventions for the treatment of metabolic diseases in overweight patients.

Regarding mechanisms, bariatric surgery-induced weight loss is an effective treatment of adiposopathy (“sick fat”). As such, understanding the improvement in adipose tissue function with bariatric surgery is critical towards understanding how and why metabolic diseases are so dramatically improved. In general, improved nutrition, increased physical activity and certain pharmaceuticals consistently improve adipose tissue endocrine and immune responses which, in turn, contributes to the improvements in T2DM, hypertension and dyslipidaemia (13) (Table 2). Similarly, weight loss from bariatric surgery may (dramatically) improve metabolic disease, which is a consistent finding across multiple clinical trials (18). Therefore, although much remains to be defined, it is reasonable to conclude that the fat weight reduction with bariatric surgery results in anatomic and physiological improvements in pathogenic adipose tissue, contributing to improvements in multiple endocrine and immune responses, which then leads to improvements in metabolic disease.

### Challenge #2: While bariatric surgery indication guidelines exist, data are not always specific as to who metabolically benefits most from these procedures

*Claim*: Currently, the criteria for bariatric surgery are based on BMI criteria. Bariatric surgery is indicated for patients with BMI > 40 kg/m^2^, or > 35 kg/m^2^ if associated with obesity-related comorbidities. Under these current guidelines, patients with BMI < 35 kg/m^2^ are not candidates for bariatric surgery.

*Response*: For the morbidly obese patient, weight loss with bariatric surgery may be helpful to treat various adverse multiple clinical consequences related to fat-mass alone, including various cardiovascular, neurological, pulmonary, musculoskeletal, dermatological, gastrointestinal, genitourinary, renal and psychological diseases (78,79). Additionally, bariatric surgery prior to pregnancy (or planned pregnancy) may improve fertility, and may have postpregnancy maternal and neonatal benefits (80,81).

However, strictly from a metabolic standpoint, not all overweight or obese patients have evidence of metabolic disease (82). In fact, some have described an ‘obesity paradox’ wherein overweight patients may have some ‘protection’ against the consequences of metabolic diseases, such as improved outcomes following atherosclerotic coronary heart disease (CHD) events (11,83). Conversely, some patients may have metabolic diseases associated with adiposity, but do not fit the BMI criteria of obesity, such as sometimes occurs with Asian Indians (1,9,11). Thus, at least from an endocrine and immune standpoint, the pathogenic potential of adipose tissue is less dependent upon the degree of increased fat mass, but instead more related to the manner in which positive caloric balance results in ‘sick fat,’ (84) and the subsequent interactions or ‘cross-talk’ that occurs between adipose tissue and other body organs (9).

Although the cut-off points for bariatric surgery are BMI > 40 kg/m^2^, or > 35 kg/m^2^ when associated with comorbidities (85), some data suggest that bariatric surgery could benefit patients with adiposopathy and BMI < 35 kg/m^2^, with significant improvement in metabolic diseases, such as those with T2DM (86). This is presumably because pathogenic adipose tissue can contribute to metabolic disease, even with only mild to modest fat weight gain, suggesting that adiposopathy might be a more rational target for treatment of the overweight patient with metabolic disease than BMI alone (84). However, the current data available on the metabolic benefits of bariatric surgery in patients with BMI < 35 kg/m^2^ are very limited. So while encouraging, more research is needed to more fully examine the spectrum of patients who might best benefit from bariatric surgical intervention.

Until selection criteria are better defined for patients who might additionally benefit from bariatric surgery based upon their metabolic risk, the challenge for clinicians is providing access to care for patients who meet existing criteria for bariatric surgery. Currently, only ∼1% of eligible candidates (based upon existing guidelines) are treated with bariatric surgery (87). This suggests that additional education is needed for clinicians who are in the best position to refer patients for bariatric surgery, as well as among obese patients who might potentially explore this option.

### Challenge #3: Bariatric surgery is too risky compared with other existing treatments for metabolic disease

*Claim*: Bariatric surgery is an extreme treatment option for treating patients with obesity, T2DM, hypertension and dyslipidaemia and has an unacceptable high degree of morbidity and mortality.

*Response*: While some healthy overweight and obese individuals may not be at increased morbidity or mortality risk, obesity is generally associated with an increase in overall mortality (88). Multiple clinical trials demonstrate that bariatric surgery in obese individual decreases overall mortality compared with control groups (89–93). Admittedly, the most landmark obesity outcomes trials are not randomised control trials, and thus their interpretation has limitations. However, the totality of data thus far suggests that bariatric surgery is the only method of intentional weight loss that may save lives, as demonstrated by large controlled clinical trials, conducted over a long period of time. Regarding safety, as with all surgical procedures, bariatric surgery does have risk (Table 1), as do pharmaceutical drug therapies (94). The degree of risk for bariatric procedures is not unlike the risks of many other surgical procedures, and is related to the surgical procedure itself (with gastric bypass having a higher rate of morbidity and mortality compared with LABP) (Table 1) as well as the expertise of those involved in conducting the procedure.

In an effort to address the need to maximise the reduction of surgical risk, ‘Centers of Excellence’ have emerged in an attempt to standardise and improve the quality of care of bariatric surgery patients. Recognition as a ‘Center of Excellence’ may be achieved by undergoing certification processes through the American Society for Metabolic and Bariatric Surgery and the American College of Surgeons (21), and adhering to peri-operative guidelines, such as those established by the American Association of Clinical Endocrinologists, the Obesity Society and the American Society for Metabolic & Bariatric Surgery (95). Through the use of experienced surgical centres, multiple studies of large numbers of patients reveal that the operative and postoperative (< 90 days) mortality of the more common bariatric procedures is < 1%, which is less than many other surgical procedures (21). This is not to say that bariatric surgery is without risk. Acute morbidities occur in ∼5–10% of patients, and chronic complications occur as well (21) (Table 1). This is also not to say that bariatric surgery is a substitute for appropriate nutrition and physical activity, which although not often successful (96), are the safest and the most cost effective approach to the individual overweight patient with metabolic disease.

Finally, it is commonplace that rather than treating the underlying cause of metabolic disease (excessive body fat leading to pathogenic adipose tissue), clinicians often utilise drug therapies specific for individual metabolic diseases that subsequently emerge in overweight and obese patients. The approach of treating the consequences of adiposopathy (rather than the cause) does have rationale, given that many anti-diabetes, anti-hypertensive and anti-dyslipidaemia agents have well-documented morbidity and sometimes mortality outcome data supporting their use. However, while they often improve metabolic disease, no anti-obesity drug therapy has proved to reduce overall mortality. By contrast, bariatric surgery not only often results in disease remission (21), but multiple studies support decreased mortality (89–93,97,98). Therefore, in making the best treatment decision, the patient and clinician must balance the potential benefits and risks of non-surgical vs. surgical options in obese patients with metabolic disease, based upon the best available data.

### Challenge #4: Different bariatric surgery procedures may have different outcomes

*Claim*: Bariatric surgical procedures vary, as does the weight loss following bariatric surgery. These differences are not always well-known among patients and clinicians.

*Response*: Although the discussions of the metabolic benefits bariatric surgery are often lumped (inappropriately) together, different bariatric surgery procedures may have different effects upon adipose tissue responses. They may therefore have different effects upon metabolic disease (Tables 1 & 2). In general, gastric bypass results in greater weight loss than LAGB over 2 years, with approximation of weight loss beyond 4 years. Conversely, LAGB has lower short-term morbidity, although possibly greater reoperation rates (16). However, as noted earlier, the morbidity and mortality of all bariatric surgeries are highly dependent upon who performs the surgery, and where the procedure is being conducted. Furthermore, any data regarding the absolute or relative rates of bariatric surgery morbidity and mortality must take into account the variable of time, in that surgical techniques are constantly advancing towards the relentless pursuit of improved outcomes (99).

The differences in these procedures with regard to the speed of initial weight loss and the physiological changes unique to gastrointestinal diversion may account for the higher rates of early improvement in metabolic diseases often observed in gastric bypass patients, compared with those treated with LAGB (21). Understanding the potential risk and benefit differences between common bariatric procedures is important for patients and clinicians before considering surgery to treat metabolic disease. For example, the potential long-term nutritional deficiencies sometimes associated with gastric bypass need to be balanced against better shorter term weight loss and reduced glucose levels in overweight patients with T2DM.

### Challenge #5: As head-to-head clinical trials are limited, the short- and long-term safety and efficacy of bariatric surgery compared with medical therapies are largely unknown

*Claim*: While the weight loss achieved with bariatric surgery is better than with anti-obesity drug therapy for most patients, isolated patients can achieve significant weight loss and weight loss maintenance, and improvement in metabolic disease with changes in their nutrition and physical activity, with or without pharmacotherapy. Furthermore, even among those who lose weight and have initial improvement in metabolic disease, many patients who undergo bariatric surgery regain weight, and potentially have recurrence of their metabolic disease status. Thus, little comparative data support bariatric surgery as a reasonable alternative to medical therapy in improving metabolic disease in overweight patients.

*Response*: The medical and surgical literature differs in the convention by which weight loss is reported. In studies of anti-obesity drug therapies, weight loss is most often described as the per cent weight loss, as expressed by a placebo-subtracted reduction in body weight from the point of randomisation. However, in the surgical literature, weight loss measurement with bariatric surgery is often described in terms of ‘excess weight,’ which is the change in body weight beyond the ideal body weight. Weight loss divided by ‘excess body weight’ is often reported as the per cent ‘excess weight loss’ or EWL. The EWL reported in bariatric surgery trials may appear to magnify the results in surgical weight loss studies if directly compared with what is reported in medical weight loss studies. Thus, the distinction between how weight loss is often reported is necessary to properly compare the efficacy of drug vs. surgical obesity treatments (100).

Another issue that complicates comparisons of anti-obesity treatments is that the largest and most compelling bariatric surgery trials report results in terms of control group comparisons, and not in terms of classic randomisation to placebo. This is because to achieve a true placebo control, sham surgery studies would have to be performed, which are generally considered unethical, not practical and thus true placebo-controlled trials are unavailable (101). Yet other challenges in making efficacy comparisons include the lack of reporting of absolute data points (with graphs being displayed instead), and lack of consistent reporting of month-by-month data time points. Additionally, weight loss clinical trials have other intrinsic inherent challenges (102). These challenges are exacerbated when trying to compare weight loss efficacy across different therapies because clinical trials often have substantial differences in study designs (e.g. some anti-obesity drug trials have elements of crossover design), variances in placebo lead-in (in anti-obesity drug trials), variances in the time of lead-in diet therapies (in anti-obesity drug trials), different entry populations, different baseline demographics (the BMI of patients in weight loss pharmacological trials are often less than the BMI of patients undergoing bariatric surgery), different research sites conducting the studies, different reporting of completers vs. intent to treat, and different number/percentage of study subject ‘drop outs.’

Regarding the durability of weight loss maintenance, this is one area in which the reported data are more robust with surgery vs. drug therapy for treatment of obesity. Bariatric surgery has published data of continued efficacy and reduced mortality data spanning as long as 10 years (14,89,90). Conversely, the published literature regarding the long-term efficacy of anti-obesity drug therapy is typically no longer than 1–2 years (although rare studies have reported 4-year results) (103). However, a perception confounder that may affect the clinician’s perspective is that individual patients may substantially vary in their maintenance of weight loss with bariatric surgery, as is true with anti-obesity drug therapies. Other perception confounders are that bariatric surgery “failures” may be remembered more by clinicians than successes, and non-surgical obesity specialists may receive more referrals of bariatric “failures” than bariatric surgery successes.

Finally, some comparative data do support that treating the underlying cause of metabolic disease (i.e. adiposity leading to adiposopathy) with bariatric surgery may be a more effective strategy to improve metabolic disease, when compared with interventions directed at treating the consequences of metabolic disease. At least one study compared LAGB vs. a multifaceted intensive medical treatment (including very low calorie diet, anti-obesity pharmacotherapy and behaviour modification) in overweight patients with BMI 30–35 kg/m^2^. (It is noteworthy that this entry BMI was below current bariatric surgery guidelines.) After 24 months, LAGB was more effective than non-surgical therapy in reducing weight, reducing metabolic syndrome and improving quality of life measurements (15). Many of these same authors also evaluated overweight T2DM patients with BMI 30–40 kg/m^2^, and compared LAGB versus a diabetes medical team recommendation of lifestyle modification (such as appropriate nutrition and physical activity), with or without other weight loss interventions (such as very low calorie diets and anti-obesity pharmacological therapies). LAGB achieved greater weight loss, improved glucose control and induced greater remission of T2DM than non-surgical, ‘conventional’ diabetes mellitus intervention (15).

### Challenge #6: Bariatric surgery may not be cost effective

*Claim*: While both might be expected to improve comorbid conditions, including metabolic diseases, bariatric surgery is much more expensive than non-surgical approaches to weight loss and is not cost effective.

*Response*: Before addressing the cost of weight loss treatment, it should be recognised that the cost of a lack of effective obesity treatment is also substantial (104). But with regard to treatment, the determination of what is ‘cost effectiveness’ is dependent upon the baseline demographic of the patient, the length of the analysis and the dollar amount per quality-adjusted life years (QALY). Economic evaluations of anti-obesity drug therapy (e.g. orlistat, sibutramine and rimonabant) suggest that such treatments are generally in the range of what is regarded as ‘cost effective’ (105). One commonly cited amount thought to represent cost effectiveness is US $50,000 per QALY (106). Using this figure, both LAGB and gastric bypass appear to be cost effective at < $25,000 each, with LAGB perhaps being more cost effective than gastric bypass (107). In another analysis of insurance claims with year of index dates ranging from 1 to 6 years, and which assumed the initial investment for LAGB being ∼$17,000 and the initial investment for gastric bypass being ∼$26,000, the health cost savings of bariatric surgery was suggested to offset the health costs in those not undergoing surgery after 2 years for laparoscopic surgery and 4 years for open surgery (108). Additional analyses further support surgically induced weight loss as cost effective in managing overweight patients with T2DM (109,110).

In summary, the cost for bariatric surgery is within the range of ‘cost effectiveness,’ with an important reason being the reduction in pharmaceutical costs in patients with baseline, drug-treated, obesity-related comorbidities (111,112). To the extent that bariatric surgery may not be ‘cost effective’ for the individual patient who experiences rare and severe complications, it is perhaps relevant that some studies suggest that LAGB is associated with less readmissions after one year compared to gastric bypass (113) while other studies suggest that gastric bypass has less incidence of readmission for reoperation (16). These rates are highly variable and depend on what surgery is performed, where the surgery is performed, who performs the surgery, the types of patients who undergo the surgery and are thus important considerations given that reoperations are an important determinate regarding long-term cost effectiveness.

### Challenge #7: Patients undergoing bariatric surgery face ‘continuity of care’ issues that may compromise their long-term prognosis

*Claim*: A treatment knowledge gap often exists after bariatric surgery is performed. It is simply reality that surgeons are not the clinicians most often involved in the long-term medical management of postbariatric surgery patients, their long-term metabolic disease management, or even the needed surveillance of potential long-term complications of bariatric surgery (Table 1).

*Response*: The need for the primary care clinician to assume medical management of the postoperative surgical patient is a practical and common scenario in the day-to-day general practice of medicine. For example, abscess lesions and surrounding cellulitis is an illustrative example of a ‘sickness’ with a defined cause (e.g. infection) with potential serious consequences (e.g. inflammation and sepsis) that is most effectively managed surgically (e.g. incision and drainage) and subsequent medical management (e.g. antibiotics). The same could be said about CHD which has a defined cause (the inflammatory process of atherosclerosis) with potential serious consequences (e.g. angina and subsequent myocardial infarction) that is often managed surgically (e.g. coronary bypass surgery), and subsequent medical management (e.g. lifestyle and pharmaceutical intervention of multiple metabolic diseases to reduce future CHD risk).

Similarly, adiposopathy has a defined cause (e.g. positive caloric balance in environmentally and genetically susceptible patients) with potential serious consequences (e.g. pathogenic endocrine and immune processes leading to metabolic disease), that in many cases, may be most effectively managed surgically (e.g. bariatric surgery) and subsequent medical management (e.g. potential adjustment of drugs used to treat T2DM, hypertension, dyslipidaemia, etc.).

Unfortunately, many clinicians are far more familiar with the partnership of surgical and medical care in the patient with a lanced abscess and coronary artery bypass, than the medical management of the postbariatric surgery patient. Many clinicians may not have confidence in their knowledge of the nuanced efficacy, safety and tolerability differences of various bariatric surgical procedures, and thus may not have confidence in their ability to appropriately recommend bariatric surgery to their patients. Many may not know how best to follow postbariatric patients for potential long-term complications (Table 1). Given that the vast majority of patients with metabolic diseases are managed by primary care clinicians and not specialists, then the clinician may conclude it is best not to refer their overweight patients for bariatric surgery, in the spirit of *primum non nocere* (first do no harm). From a practical standpoint, the development of a new specialisation in ‘bariatric medicine’ will hopefully help improve the care of obese patients. However, it is unlikely that bariatric medicine will overcome the need for greater awareness and understanding of bariatric surgery by primary care clinicians. Thus, the unfamiliarity of the totality of safety and efficacy issues with bariatric surgery among clinicians remains a challenge and potential impediment for the expanded use of bariatric surgery for patients with adiposopathy and metabolic disease.

It is with this challenge in mind that this review was written for the purpose of improving clinician understanding of the improvement in adiposopathy with bariatric surgery, and the subsequent improvement in metabolic diseases through improvement in adipose tissue functionality. Other publications and statements by various governmental and medical societies provide a more extensive and global discussion of the clinical management aspects of bariatric surgery as a treatment for severe obesity. Examples include the US Department of Health and Human Resources (114) and consensus conference statement by the American Society for Bariatric Surgery and the American Society for Bariatric Surgery Foundation (115). Another resource that provides clinical practice guidelines (CPG) includes the American Association of Clinical Endocrinologists, The Obesity Society, and American Society for Metabolic & Bariatric Surgery publication entitled: Medical Guidelines for Clinical Practice for the Perioperative Nutritional, Metabolic and Non-surgical Support of the Bariatric Surgery Patient, which states:

*“These CPG will focus on the non-surgical aspects of perioperative management of the bariatric surgery patient, with special emphasis on nutritional and metabolic support”.* The organization of these GPG is as follows: (i) an ***Introduction*** to familiarize the reader with the principles of bariatric surgery, (ii) a ***Methods*** section to outline the *a priori* evidence-based system of recommendations, (iii) an ***Executive Summary*** section of specific, practical evidence-based recommendations, (iv) an ***Appendix*** section containing in depth discussion and ratings of the clinical evidence referred to in the Executive Summary of Recommendations, and lastly (v) an extensive ***Reference*** section in which each clinical report or study is assigned an evidence level. (22)

Finally, it is the increasing recognition that bariatric surgery not only improves the weight of patients, but also the metabolic health of patients that prompted the ‘Bariatric Surgical Society’ to change its name to the ‘American Society for Metabolic & Bariatric Surgery’ (116). It is also the recognition that improving the health of patients with bariatric surgery is largely dependent upon meeting quality standards that has prompted evidenced-based recommendations for ‘Best Practices’ in weight loss surgery (117–129).

### Challenge #8: Bariatric surgery sends the ‘wrong’ message to obese patients regarding the importance of needed lifestyle changes and too many patients consider it a ‘quick fix’

Claim: Society cannot continue to develop, and utilise risky and expensive interventions to treat what is a voluntary, lifestyle problem. Bariatric surgery is not justified, even in patients who might reasonably be expected to have improvement in metabolic disease, because gaining excessive body fat is not because of a ‘disease,’ but rather because of poor personal behaviour. By offering bariatric surgery as a treatment option to overweight patients with metabolic disease, patients may perceive the clinician as arguing against appropriate nutrition and physical activity as the most appropriate first step in achieving weight reduction and in improving their metabolic disease. Improved nutrition and increase physical activity are far more appropriate and much more cost effective for individual overweight patients with metabolic disease.

*Response*: Bariatric surgery is not a panacea. A good faith effort at behaviour modification, medical nutritional therapy, appropriate physical activity and pharmacotherapy should be implemented first, with consideration of bariatric surgery being reserved for obese patients who have failed medical management. However, even when indicated, bariatric surgery should not be considered a ‘cure’ for obesity and adiposopathy, but rather a tool that will help patients control their calorie intake with the objective of significant and sustained weight loss and improvement of comorbidities. Therefore, prior to recommending bariatric surgery, the clinician should ensure that the obese patient has undergone thorough screening for the surgical procedures, and education about the surgical procedures. After bariatric surgery, patients need to adhere to appropriate nutrition and physical activity to maximise weight loss benefits, such as improvements in adipose tissue function and maintenance of improved metabolic disease. Patients may also benefit from additional measures, such as support groups. But even when obesity is largely caused by poor lifestyle habits, the withholding of available safe and cost-effective therapy cannot be justified. This holds true even if the treatment is surgery – especially among obese patients with adiposopathy who may be expected to have substantial improvement, if not remission, in obesity-related metabolic diseases.

## Conclusion

Bariatric surgery is an effective and generally safe method for the treatment of obesity that often results in long-term weight loss and improvement and/or remission of metabolic diseases and comorbidities. In selective obese patients with metabolic disease who are refractory to medical management, adiposopathy (“sick fat”) is a surgical disease.
